# Beyond mortality: A fungicide mixture alters physiology and causes impaired behavior in zebrafish

**DOI:** 10.1007/s10646-026-03147-z

**Published:** 2026-09-01

**Authors:** Aline Pompermaier, Flavia Bernardo Chagas, Wagner A. Tamagno, Leandro Galon, Paulo A. Hartmann, Marilia Hartmann

**Affiliations:** 1 Laboratory of Ecology and Conservation, Federal University of Fronteira Sul, Erechim, RS Brazil; 2 School of Health Sciences, Purdue University, West Lafayette, IN USA; 3 Federal University of Fronteira Sul, Erechim, RS Brazil

**Keywords:** Behavior, Fish, Fungicide mixtures, Non-target organisms, Pesticides, Shoaling

## Abstract

Pesticides have been widely used to ensure high productivity in monoculture systems. However, due to pathogen resistance and the need to improve the safety and sustainability of these substances, new compounds have emerged on the market. One example is pesticide mixtures, which aim to provide a broader spectrum of functionality within a single product. The azoxystrobin- and tebuconazole-based fungicide (ATBF) is a commercial premix fungicide containing two active ingredients. Nevertheless, studies investigating the effects of such mixtures on non-target organisms remain a significant gap in literature. Therefore, the main goal of this study was to investigate the toxicological effects of this fungicide mixture on the physiology and behavior of zebrafish. Developmental exposure (3–120 h post-fertilization [hpf]) to environmentally relevant concentrations was evaluated through assessments of survival, hatching, spontaneous movement, heart rate, and behavior (shoaling and antipredator response). Exposure to ATBF impaired survival, reduced spontaneous movement, altered heart rate, and affected shoaling behavior in zebrafish larvae. These findings highlight the importance of research on pesticide mixtures, as these products may exert additive or synergistic effects on aquatic fauna.

## Introduction

A wide range of products is available on the agricultural market to control agricultural pests, including herbicides, insecticides, and fungicides (Agrofit 2025). However, the current high demand for food and increasing pest resistance have driven the chemical industry to accelerate the development of new molecules and mixtures capable of controlling a broader range of pathogens.

In this context, Brazil is one of the largest consumers of pesticides in the world, with record numbers of approved substances in recent years (Mapa 2021; Ibama 2024). This intensive use makes the presence of these substances in water resources inevitable (Munaron et al. 2023; Panis et al. 2024), and studies have detected multiple pesticide molecules and mixtures in aquatic environments (Schemmer et al. 2024).

The purpose of these mixtures is to increase efficacy, reduce the number of applications, and improve product stability and safety. This approach addresses several challenges faced by the agricultural sector, such as pathogen resistance (Queirós et al. 2023; Siddiqui et al. 2023), operational efficiency through reductions in preparation and application time and costs, safety by minimizing the risk of chemical incompatibilities and dosing errors, and sustainability through integrated disease management and reduced environmental impact. In addition to these sector-specific challenges, climate change can alter the global distribution of pests and their resistance to pesticides (Ma et al. 2021), further highlighting the relevance of these investigations in light of recent global events driven by climate change.

However, because these products are relatively new to the market, few studies have investigated their effects on non-target organisms, leaving the final challenge identified in agriculture, sustainability, still unresolved. The azoxystrobin- and tebuconazole-based fungicide (ATBF) is an interesting candidate for such investigations. It consists of a mixture of active ingredients recommended for fungal disease control in numerous crops, including cotton, garlic, peanuts, irrigated rice, oats, bananas, potatoes, coffee, sugarcane, canola, onions, carrots, barley, citrus, beans, sunflowers, mangoes, melons, millet, corn, soybeans, sorghum, staked tomatoes, industrial tomatoes, wheat, triticale, and grapes (Adama 2022). This mixture helps control both fungal growth and spore germination and has been widely used because of its high efficacy (Bhullar and Mahajan 2025). For this reason, ATBF compounds are produced and sold to farmers as commercial premixes in several countries, including Australia, Argentina, Canada, Chile, Spain, Germany, Peru, and Brazil (e.g. Adama, 2025).

In Brazil, ATBF is classified as toxicity category IV (slightly toxic), whereas its environmental hazard potential is classified as Class II (highly dangerous to the environment) (Agrofit 2025). Its mechanism of action targets respiration and sterol biosynthesis in fungal membranes, belonging to Fungicide Resistance Action Committee (FRAC) Groups C3 and G1, inhibiting energy production (mitochondrial respiration) and compromising fungal survival (Adama 2022). It is important to emphasize that product application may be performed by either ground or aerial spraying, increasing the likelihood of reaching aquatic environments.

The effects of pesticide mixtures on non-target organisms, particularly fish, have been reported in recent studies. A mixture of chlorpyrifos, malathion, carbendazim, and atrazine, even at low concentrations, altered the antioxidant system of tambaqui (*Colossoma macropomum*) (Souza et al. 2024). A mixture of imidacloprid and propoxur at environmentally relevant concentrations induced oxidative stress and cholinergic effects in the neotropical fish *Rhamdia quelen* (Marins et al. 2021). Severe genotoxic effects were observed in *Oreochromis niloticus* exposed to a mixture of chlorpyrifos, endosulfan, and bifenthrin (Ambreen et al. 2023). A mixture of azoxystrobin and cyproconazole led to bioaccumulation, neurotoxicity, and alterations in oxidative stress biomarkers in *Prochilodus lineatus* (Rossi et al. 2024). Behavioral effects were reported in zebrafish exposed to a mixture of glyphosate and fipronil (Chaulet et al. 2019) and to a mixture of 2,4-D and imidacloprid (Siqueira et al. 2024).

However, studies evaluating the effects of azoxystrobin and tebuconazole remain scarce. In the case of azoxystrobin, parental exposure caused developmental effects and disrupted gene expression in F1 zebrafish embryos (Cao et al. 2019a), induced genotoxicity in *Pethia conchonius* (Ray et al. 2024), and generated oxidative stress in zebrafish (Jia et al. 2018). It has also been shown to induce developmental effects in both larval and adult zebrafish, including mitochondrial dysfunction, oxidative stress, cellular apoptosis, and activation of innate immune responses (Cao et al. 2018), as well as delaying sexual development and altering reproduction in the same species (Cao et al. 2019b).

For tebuconazole, various adverse effects have been reported in fish, including oxidative stress, endocrine disruption, histopathological alterations, neurotoxicity, and behavioral changes (Carnib et al. 2025). Moreover, a review study highlighted the toxic effects of tebuconazole on humans, mammals, aquatic organisms, soil animals, amphibians, soil microorganisms, birds, bees, and plants, affecting multiple endpoints including developmental toxicity, genotoxicity, reproductive toxicity, mutagenicity, hepatotoxicity, neurotoxicity, cardiotoxicity, and nephrotoxicity (Dong 2024). The same study emphasized the need for further research on the toxicity of tebuconazole mixtures. It is important to note that, unlike previous studies in which compounds were experimentally mixed for testing, the present study uses a commercial premix product originally formulated as a combination of azoxystrobin and tebuconazole. However, the increasing use of premixes in agriculture requires ecotoxicological investigation, as these compounds in combination may be highly harmful, although the extent of their effects remains unknown.

Zebrafish (*Danio rerio*) were selected as the animal model because of the advantages offered by their embryonic stage for investigating contaminant-induced effects during early development. This is facilitated by embryo transparency and external fertilization (Kimmel et al. 1995), allowing direct observation of physiological changes. Additionally, rapid embryonic and larval development enables analyses within short timeframes, with larvae exhibiting species-specific behaviors within only a few days after fertilization (Clift et al. 2014).

Therefore, this study investigated the effects of exposure to environmentally relevant concentrations of an azoxystrobin- and tebuconazole-based fungicide mixture (ATBF), a systemic product belonging to the strobilurin (azoxystrobin) and triazole (tebuconazole) chemical groups, on survival, hatching, spontaneous movement, heart rate, and shoaling and antipredator behaviors in zebrafish embryos and larvae. This study provides a novel contribution to ecotoxicology by investigating the effects of a commercially formulated fungicide and addressing an important knowledge gap, as toxicological studies are still predominantly based on isolated active ingredients or experimentally prepared mixtures.

## Materials and methods

### Study strategy

To evaluate the effects of ATBF on zebrafish development, embryos and larvae were exposed to ecologically relevant concentrations (30, 100, or 180 µg L⁻¹) or a negative control during the organogenesis phase (3–120 h post-fertilization [hpf]), followed by the evaluation of developmental parameters, shoaling behavior, and antipredator responses.

### Reproduction

For embryo collection, healthy wild-type zebrafish (*Danio rerio*, strain 5D) aged 6–18 months and maintained at the Ecology and Conservation Laboratory of the Federal University of Fronteira Sul, Erechim campus, Brazil, were placed in dedicated breeding tanks on the afternoon before spawning. Embryos were obtained from four breeding tanks, each containing five females and eight males. The following morning, one hour after the lights were turned on, embryos were collected and rinsed with E3 medium (reverse osmosis water + 60 mg L⁻¹ Ocean Tech Bio Active^®^, Hong Kong, China) to remove debris. They were then assessed for fertility and potential malformations. Embryos from all breeding tanks were pooled before allocation to the experimental treatments to minimize parental effects and increase genetic diversity within the experimental population.

### Exposure and maintenance of embryos and larvae

For the exposures, a commercial azoxystrobin- and tebuconazole-based fungicide premix (ATBF) containing 120 g L⁻¹ azoxystrobin, 200 g L⁻¹ tebuconazole, and 764 g L⁻¹ other ingredients (excipients) was used. The product is registered by the Brazilian Ministry of Agriculture, Livestock and Supply (MAPA) under registration number 13,612. The selected exposure concentrations were based on environmental detections of tebuconazole (30 and 100 µg L^− 1^; Pinheiro et al. 2010; Brovini et al. 2023) and the maximum allowable limit of tebuconazole for drinking water in Brazil (180 µg L^− 1^; Ministry of Health, 2021), and are below the average (1,055 µg L^− 1^) and maximum (6,672 µg L^− 1^) values detected for axozystrobin (Corrêa et al. 2022).

Considering that the tested product is a mixture of two active ingredients, the exposure concentrations were defined based on the concentration of the component present at the highest proportion, tebuconazole (200 g L⁻¹). From these values, the corresponding nominal test concentrations were calculated. Because the formulation also contains azoxystrobin (120 g L⁻¹), the equivalent concentrations of this compound were estimated and are presented in Table 1. Serial dilutions were performed, starting from an initial stock solution of 1 g L⁻¹, followed by an intermediate stock solution of 0.01 g L⁻¹, and finally the preparation of the test solutions. All solutions were prepared in E3 medium.

**Table 1:** Nominal test concentrations of the azoxystrobin- and tebuconazole-based fungicide (ATBF) and the corresponding equivalent concentrations of its active ingredients

Product concentration (µg L⁻¹)	Tebuconazole (µg L⁻¹)	Azoxystrobin (µg L⁻¹)
30	30	18
100	100	60
180	180	108

Embryos were exposed during the organogenesis period (3–120 hpf; Pompermaier et al. 2022) in 24-well cell culture plates, with each well containing 3 mL of test solution or E3 medium (negative control) and 10 embryos. After 120 h of exposure, the test solutions were removed, and the animals were transferred to new 24-well culture plates containing only E3 medium until the end of the experimental protocol (168 hpf). Plates were maintained in a water bath at 28 °C, and embryos were kept under a 12 h light:12 h dark photoperiod.

### Analysis of embryos and larvae

#### Survival and hatching

Mortality was recorded daily for seven days. Animals showing no touch response and displaying coagulation were considered dead and removed from the wells. Hatching was monitored until 72 hpf. Embryos were considered hatched when they had either completely ruptured the chorion or had all or most of the tail extruded from the egg. To assess survival and hatching, we monitored 110 embryos and larvae per group.

#### Spontaneous movement and heart rate

At 24 hpf, spontaneous movement (SM) was recorded for 60 s in 20 embryos per group (*n* = 20) using a stereomicroscope (Olympus SZ51). At 72 hpf, heart rate (HR) was assessed by counting heartbeats for 60 s in 20 larvae per group (*n* = 20) using a binocular biological microscope (Olympus CX21FS1).

### Behavioral analysis

#### Shoaling test

To determine whether ATBF exposure affects shoaling behavior in zebrafish larvae, shoaling tests were conducted at 120 hpf, adapted from Tamagno et al. 2026. The test was performed in 6-well cell culture plates (15 mL capacity) filled with E3 medium, with four larvae per well. A total of 12 wells per treatment group were evaluated, resulting in 48 larvae per tested concentration (*n* = 12).

Larvae were video-recorded for six minutes using an iPhone 11 camera (iOS 18) positioned above the testing arena. For analysis, screenshots were extracted every 30 s from each recording, yielding 12 images per well. For each image, the distances between larvae within each well were measured and used to calculate the mean inter-larval distance. These mean distances were subsequently compared among experimental groups. Measurements were performed using ImageJ software. The mean value obtained from all images of a given well was considered a single independent replicate for statistical analysis.

#### Aversive stimuli test (AST)

To evaluate whether ATBF exposure impaired the antipredator response of larvae, an Aversive Stimulus Test (AST) was conducted. The assay consisted of placing 168 hpf larvae in 6-well cell culture plates (15 mL capacity) containing 10 mL of E3 medium at a density of five larvae per well, with 12 replicates per treatment group (60 larvae per treatment group, *n* = 12).

The plates were positioned above an LCD monitor and larvae were acclimated for two minutes before exposure to a visual stimulus consisting of a 1.35 cm red sphere moving along a trajectory covering half of the well area. The sphere movement was generated using PowerPoint and lasted five minutes. The percentage of larvae remaining in the stimulated area at the end of the test was calculated. Each well generated a single percentage value and was considered the experimental unit for statistical analysis. All recordings were made using an iPhone 11 camera (iOS 18). This test was adapted from Kalichak et al. 2017.

### Statistics

Survival analysis was performed using the Kaplan–Meier method, and differences among groups were evaluated using the log-rank (Mantel–Cox) test. Hatching data were analyzed using linear regression. SM, HR, AST, and shoaling data were evaluated for normality and homoscedasticity using the Kolmogorov–Smirnov and Brown–Forsythe tests, respectively. As all datasets met the assumptions for parametric analyses, data transformation was not required. Data were subsequently analyzed using one-way ANOVA followed by Dunnett’s post hoc test, with multiple comparisons performed against the negative control group. Statistical significance was established at α = 0.05. All statistical analyses were performed using GraphPad Prism^®^ version 8.01 (GraphPad Software, San Diego, CA, USA).

## Results

### Survival and hatching

**Fig. 1:**
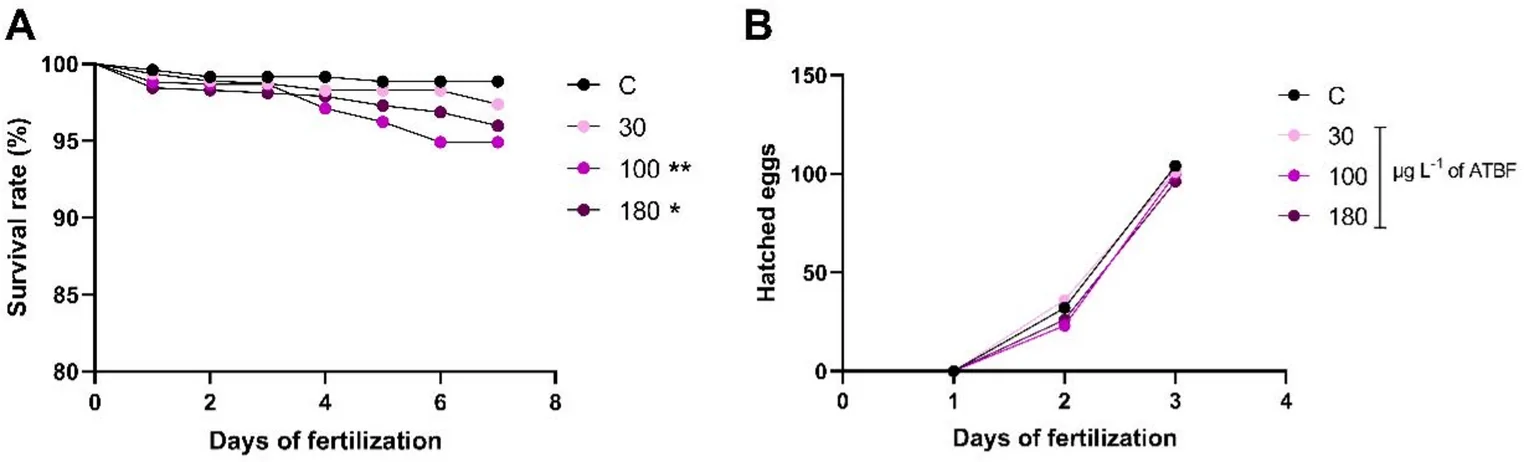
Survival rate (**A**) and hatched eggs (**B**) of zebrafish exposed to different concentrations of azoxystrobin-tebuconazole-based fungicide (ATBF). Data represent the percentage of surviving individuals over time. Survival distributions were compared using the log-rank (Mantel–Cox) test following Kaplan–Meier estimation. Asterisks indicate that the specific mortality curve of exposed embryos was different from the control embryo curve (* *p* < 0.05; ** *p* < 0.001; *n* = 110)

Survival analysis using the Kaplan–Meier method revealed significant differences in survival among treatments (χ² = 10.08, df = 3, *p* = 0.0179; log-rank test, Fig. 1A). Pairwise log-rank (Mantel–Cox) comparisons showed that exposure to the 100 and 180 µg L⁻¹ groups resulted in significantly lower survival compared with the control group. Specifically, survival in the 100 µg L⁻¹ group was reduced relative to the control (χ² = 8.583, df = 1, *p* = 0.0034), with a hazard ratio (HR) of 0.30 (95% CI: 0.15–0.62), indicating a 70% lower probability of survival compared with the control. Similarly, the 180 µg L⁻¹ group showed a marked decrease in survival (χ² = 5.572, df = 1, *p* = 0.0183), with an HR of 0.37 (95% CI: 0.18–0.80), indicating a 63% lower probability of survival compared with the control, but did not affect embryo hatching (Fig. 1B).

### Spontaneous movement and heart rate

**Fig. 2:**
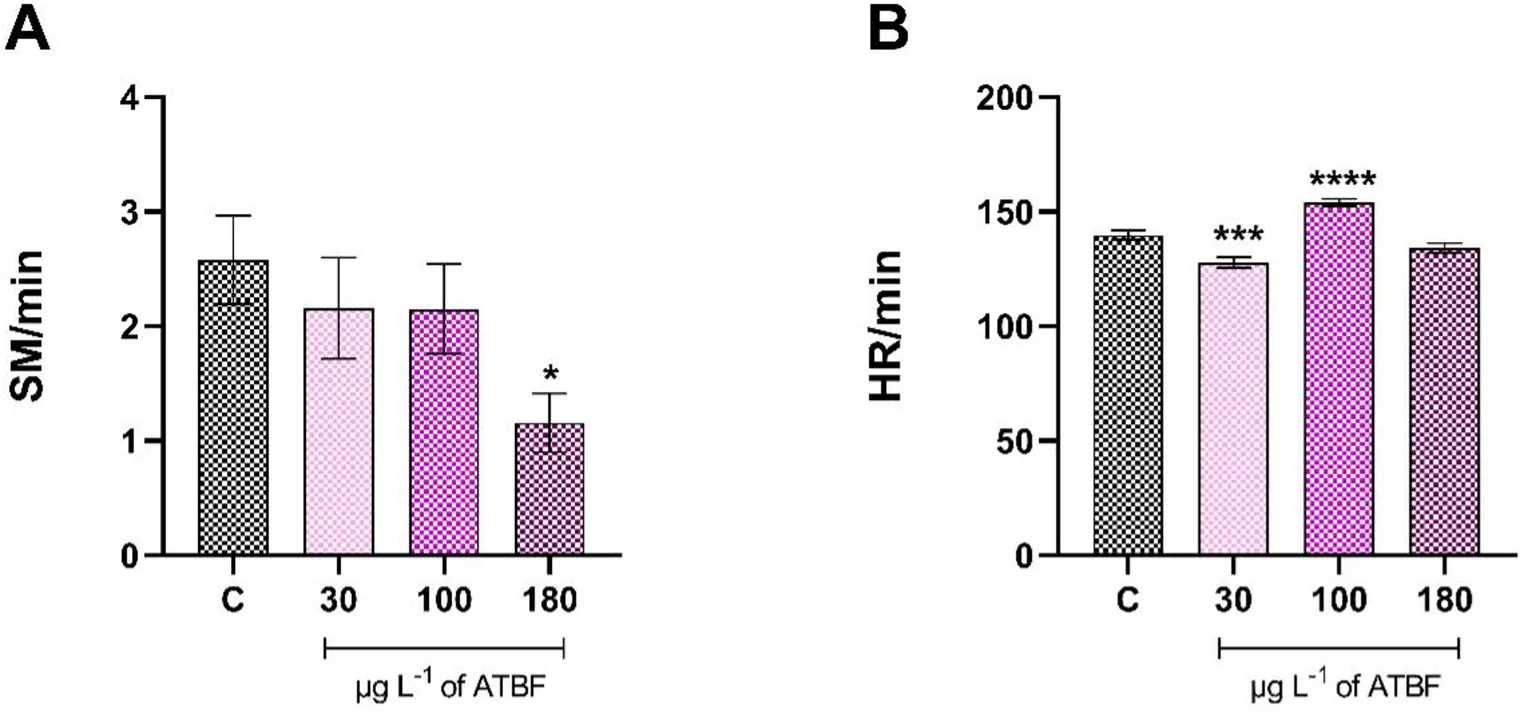
Spontaneous movements (SM, **A**) at 24 hpf and heart rate (HR, **B**) at 72 hpf in zebrafish larvae exposed from 3–120 hpf to azoxystrobin-tebuconazole-based fungicide (ATBF). SM and HR were analyzed by one-way ANOVA followed by Dunnet’s multiple comparison test. Data is expressed as mean ± SEM. Asterisks indicate statistical differences from the control group (* *p* < 0.05; ***p < 0.001; ****p < 0.0001; *n* = 20)

Exposure to ATBF decreased spontaneous movement at a concentration of 180 µg L⁻¹ (*p* = 0.05; Fig. 2A). Heart rate was reduced at 30 µg L⁻¹ but increased at 100 µg L⁻¹ (*p* < 0.0001; Fig. 2B).

### Behavioral analysis

**Fig. 3:**
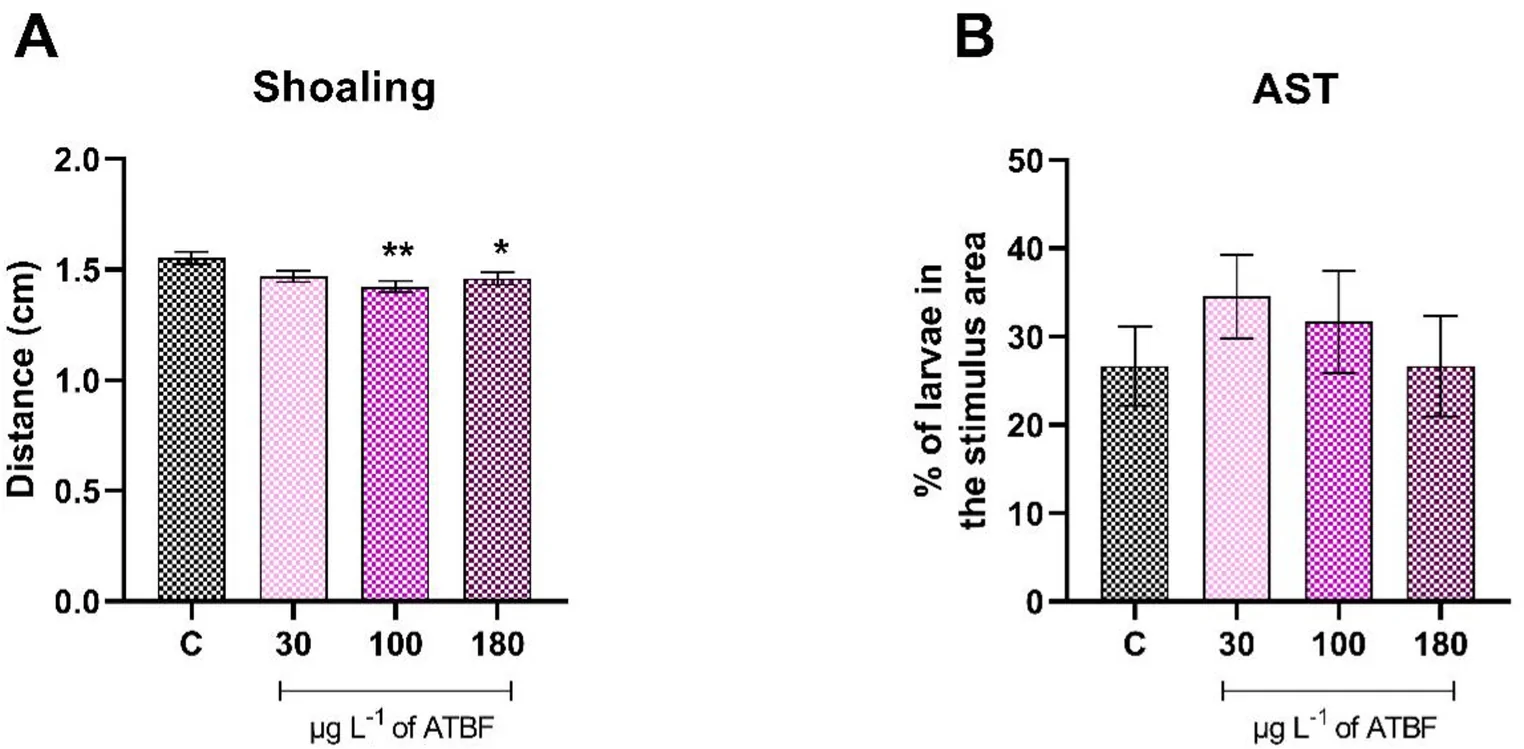
Shoaling (**A**) at 120 hpf and aversive stimulus (AST, **B**) at 168 hpf in zebrafish larvae exposed from 3–120 hpf to azoxystrobin-tebuconazole-based fungicide (ATBF). Data from panels A and B were analyzed by one-way ANOVA followed by Dunnet’s multiple comparison test and data are expressed as mean ± SEM. Asterisks indicate statistical differences about the control group (* *p* < 0.05; **p < 0.001; *n* = 12)

Exposure to ATBF increased shoal cohesion at concentrations of 100 and 180 µg L⁻¹ (*p* = 0.0056; Fig. 3A), as indicated by the reduced distance between conspecifics within the shoal. However, no effects were observed on risk perception in the AST (*p* = 0.6532; Fig. 3B).

## Discussion

Here it is shown that exposure to ATBF, a premix fungicide formulated with two active ingredients (azoxystrobin and tebuconazole), impaired survival, reduced spontaneous movement, altered heart rate, and shoaling interaction in zebrafish larvae exposed from 3 to 120 hpf. These findings are highly relevant given the intensive use of fungicide formulations in modern agriculture and the potential contamination of water bodies through leaching and natural degradation processes.

Exposure to ATBF caused mortality in animals exposed to the highest concentrations (100–180 µg L⁻¹). This effect is well documented for both commercial formulations and the active ingredient tebuconazole in various exposure scenarios (Carnib et al. 2025), as well as for azoxystrobin exposure (Jia et al. 2018). Similar effects on survival have been reported for numerous pesticides, such as glyphosate (Pompermaier et al. 2022), diquat (Pompermaier et al. 2025), and diflubenzuron, pyriproxyfen, and their mixtures (Teixeira et al. 2024). Furthermore, the concentrations tested here are environmentally relevant and raise significant concerns, as they have been detected in water resources (Pinheiro et al. 2010; Brovini et al. 2023) and may even be present in drinking water (Ministry of Health, 2021). However, when considering azoxystrobin, this becomes an even greater concern given the lack of regulations regarding permissible levels in drinking water, representing a real threat to the survival of aquatic animals under natural conditions. The mortality levels observed in this study highlight the urgent need for improved water quality monitoring and regulatory updates, as reduced survival can trigger serious ecological consequences, including disruptions in trophic cascades.

Another key parameter evaluated was spontaneous movement, which was affected by the highest tested concentration (180 µg L⁻¹). At this concentration, the animals exhibited a reduced number of movements throughout the test, which may serve as an early warning sign of neurotoxicity and overall developmental impairment. Indeed, spontaneous movement in embryos is controlled by the developing nervous system (Saint-Amant and Drapeau 1998). Alterations in this activity strongly suggest that the contaminant may be interfering with neural function. In addition, it is hypothesized that this reduction in movement could be a secondary consequence of overall compromised development, as all parameters evaluated here were altered by ATBF exposure.

Intriguingly, larvae exposed to ATBF also exhibited alterations in heart rate. Exposure to 30 µg L⁻¹ decreased heart rate, whereas exposure to 100 µg L⁻¹ increased it. This may represent a biphasic or non-monotonic response, highlighting a significant effect of ATBF exposure, given that heart rate reflects physiological status (Agathokleous 2022). The mechanisms underlying this response were not investigated in the present study. Therefore, it is not possible to determine whether the observed alterations were associated with adaptive physiological responses, toxic effects, oxidative stress, or other pathways involved in cardiac regulation. Future studies should investigate the molecular and physiological mechanisms associated with these cardiac responses, including biomarkers of oxidative stress, antioxidant defenses, and signaling pathways potentially involved in cardiovascular function. Furthermore, while the present study focused on survival, hatching, and cardiac responses, the evaluation of morphological abnormalities was beyond its scope. Future studies should include morphological endpoints to provide a more comprehensive assessment of the developmental toxicity of ATBF in zebrafish embryos.

Regarding behavioral analyses, exposure to ATBF altered the social dynamics of larval groups in the shoaling test, although it did not impair their antipredator capacity in the AST. In fact, larvae exposed to the highest concentrations (100–180 µg L⁻¹) exhibited increased cohesive behavior within shoals, with a smaller average distance between individuals compared to the control group. This may indicate a potential anxiety-like state or heightened baseline risk perception (Franks et al. 2018). A well-documented behavior of zebrafish is their strong social tendency to form cohesive shoals, which represents a key antipredator strategy (Buske and Gerlai 2010). These findings suggest that ATBF exposure may have induced a state of constant alertness, causing the larvae to behave as if a predator were consistently present, even in its absence. This result, together with the unchanged response to predatory stimulation in the AST, indicates that the animals were not paralyzed or suffering from severe motor deficits. They were able to perceive the stimulus and respond to it, a reaction that has been impaired in other pesticide exposure studies (Bridi et al. 2017; Pompermaier et al. 2022). This strengthens our interpretation that the alteration is not due to an inability to react, but rather to changes in spontaneous behavior, possibly linked to dysregulation of neurotransmitters associated with anxiety and sociability.

From an ecological perspective, these findings have profound implications. An animal in a perpetual state of alert expends considerable energy, diverting resources away from processes such as growth and reproduction and potentially neglecting essential activities such as foraging and mating. Moreover, an overly cohesive and anxious shoal may become more conspicuous to predators or less efficient in locating resources, thereby increasing survival risks. Innate behavior is crucial for avoiding such risks in natural settings.

With this broad range of findings (impaired survival, reduced spontaneous movement, disturbances in heart rate, and behavioral alterations), it is important to consider the mechanisms of action involved in these effects. Here, no biochemical assessment of the embryos was performed; however, based on the literature, it is possible to better understand the toxicological profiles of the evaluated pesticides. For azoxystrobin, it has been reported to interact with enzymes of the cholinergic and antioxidant systems, resulting in marked increases in reactive oxygen species (ROS) levels, lipid peroxidation (MDA), catalase (CAT) activity, and acetylcholinesterase (AChE) activity, despite inhibition of superoxide dismutase (SOD) activity, glutathione peroxidase (GPx) activity, and acetylcholine (ACh) concentrations in the brains of male zebrafish (Guo et al. 2024). For tebuconazole, exposure is known to cause inhibition of AChE activity in zebrafish larvae (Altenhofen et al. 2017), modulation of SOD activity in zebrafish (Macirella et al. 2022), oxidative stress induction in *Cyprinus carpio* (Toni et al. 2011), and oxidative stress induction and behavioral alterations in zebrafish (Vieira et al. 2022). These alterations may be key contributors to the effects observed here following embryonic exposure to the mixture of these compounds. An interesting effect reported in the literature is the alteration of AChE activity, which is directly linked to behavioral effects (Pompermaier et al. 2022) and may indicate a neurotoxic effect of exposure. This enzyme plays a critical role in neural circuits involved in learning, memory, and motor coordination (Aroniadou-Anderjaska et al. 2023). Based on this, the increased cohesion observed in exposed larvae might be the result of dysregulation of cholinergic pathways. Although some potential mechanisms underlying ATBF-induced toxicity are discussed here, the present study did not evaluate biochemical or molecular biomarkers. Therefore, these mechanisms should be considered plausible hypotheses rather than confirmed pathways. Moreover, future studies should incorporate biomarkers such as AChE activity, antioxidant enzymes, lipid peroxidation, ROS production, and gene expression analyses to better elucidate the mechanisms involved.

Based on this information, this mixture of compounds simultaneously impacted multiple systems, leading to neurotransmitter dysregulation associated with behavioral impairments, as well as disruption of redox homeostasis and cardiac function. This dual pathway of action, neural and oxidative, may interact and potentially overwhelm the defense mechanisms of embryos and larvae, explaining the synergy and severity of the lethal and sublethal effects observed at environmentally relevant concentrations. Thus, findings from the literature not only corroborate the present results but also provide a mechanistic model for understanding the toxicity of this mixture.

It is important to note that, in this study, the product was tested as a mixture from its origin, which differs from other studies in the literature where products were mixed solely for experimental purposes. These findings further reinforce the need for additional investigations, considering that the exposures conducted here involved environmentally relevant concentrations of a commercial product that caused adverse effects in all evaluated parameters in aquatic organisms, even during early developmental stages. The impairment of survival, physiology, and behavior suggests ecological risks that extend beyond the acute toxicity traditionally evaluated in regulatory processes. In this sense, the results reinforce the need to include sublethal endpoints and behavioral analyses in environmental risk assessments, in addition to considering the effects of mixtures of active ingredients present in commercial formulations. Considering the regulatory processes for these substances, the evidence presented here highlights the need to review safety criteria and improve public policies aimed at protecting aquatic ecosystems, especially in light of the intensive use of pesticides and the fact that the concentrations tested here have been detected in the environment and are permitted for human consumption.

## Conclusion

Developmental exposure to environmentally relevant concentrations of ATBF, a premix fungicide formulated with two active ingredients (azoxystrobin and tebuconazole), impaired survival, reduced spontaneous movement, altered heart rate, and increased shoal cohesion in zebrafish larvae. Our findings highlight the importance of research on mixtures, especially premixes that enter the environment as ready-made chemical cocktails. We know very little about the individual effects of pesticides, yet it is also essential to understand how they affect organisms when combined in premix formulations.

Collectively, our results demonstrate that commercial pesticide mixtures designed to broaden the spectrum of action can pose a substantial ecotoxicological risk to non-target organisms, even at low concentrations. Our study underscores the need to expand and deepen research on mixed formulations, as active ingredients may interact in an additive or synergistic manner, potentiating toxicity to aquatic fauna. Therefore, environmental risk assessment and regulatory frameworks must evolve to consider not only active ingredients in isolation but also the integrated effects of commercially formulated products, ensuring more robust and realistic ecosystem protection.

## Data Availability

The authors declare that the data supporting the findings of this study are available within the paper. Should any raw data files be needed in another format they are available from the corresponding author upon reasonable request.
